# Physical literacy, sense of belonging in the sports environment, and college students’ mental health: an analysis of relational paths based on differences in exercise groups

**DOI:** 10.3389/fpsyg.2026.1896855

**Published:** 2026-08-04

**Authors:** Zhenlei Zhao, Linfeng Xue

**Affiliations:** 1 School of Physical Education, Hunan City University, Yiyang, Hunan, China; 2 Department of Physical Education, Communication University of Zhejiang, Hangzhou, China

**Keywords:** college students, exercise group, GLTEQ, mediating effect, mental health, perceived physical literacy, sense of belonging in the sports environment

## Abstract

**Objective:**

This cross-sectional study investigated the cross-sectional associations among college students’ perceived physical literacy, sports-environment belongingness, and mental well-being. It explored whether belongingness statistically mediated the physical literacy–well-being link, whether exercise groups (classified via Godin-Shephard Leisure Score) moderated the belongingness–well-being relationship, and examined interactive associations of high school and university sports participation.

**Methods:**

During the fall of 2025 (September to November), an online survey yielded 890 valid responses. Participants completed the Perceived Physical Literacy Instrument, Sports-Environment Belongingness Scale, WHO-5 Well-Being Index, and Godin-Shephard Physical Activity Questionnaire. Data were analyzed using OLS regression, Bootstrap-based mediation, moderation models, and two-way ANOVA.

**Results:**

Controlling for academic stress and body mass index, perceived physical literacy was positively associated with mental well-being (*b* = 0.360, *p* < 0.001). Sports-environment belongingness served as a partial statistical mediator in this link (indirect estimate = 0.169, 95% CI [0.131, 0.209]), accounting for 46.9% of the total association. Exercise group did not moderate the belongingness–well-being association (*p* = 0.900), nor did past and current sports participation present interactive associations. Sensitivity analysis indicated that including mild-intensity activities substantially increased the proportion of students classified as physically active.

**Conclusion:**

Perceived physical literacy is positively related to college students’ mental well-being, a relationship partially explained by sports-environment belongingness. These cross-sectional findings underscore the value of inclusive, psychologically safe sports environments, reflecting statistical associations rather than causality.

## 1 Introduction

### 1.1 Realistic background: mental health challenges of contemporary college students

The college stage is an important period for individuals transitioning from adolescence to early adulthood. Students usually need to simultaneously adapt to multiple tasks such as lifestyle changes, reconstruction of interpersonal relationships, academic evaluation pressure, and uncertainty about future development. The World Health Organization points out that mental health issues have become an important challenge jointly faced by the global public health and education systems, and youth and college student populations require particular and sustained attention (World Health Organization, 2022). College students, representing late adolescents and emerging adults, are at a critical developmental intersection. Their campus physical education (PE) experiences are the direct culmination of their K-12 school PE trajectories. Therefore, examining this population provides vital insights into how earlier school-based inclusivity efforts translate into lifelong physical activity habits and mental health outcomes. Therefore, how to identify and promote college students’ mental health in the university context has become a critical issue of common concern in the fields of education, psychology, and public health.

Among the many ways to promote college students’ mental health, physical activity and sports participation are considered to have significant value. Existing reviews and empirical studies generally show a positive association between physical activity and college students’ mental health, emotional regulation, and subjective well-being (Li et al., 2023; Malagodi et al., 2025; Talapko et al., 2021; Zhong, 2024). However, the association between physical activity and mental well-being may not depend solely on exercise frequency, duration, or intensity. In reality, even if students participate in similar physical education courses or campus sports, their psychological experiences and health benefits may differ significantly: some students can gain a sense of competence, pleasure, and social connection from sports activities, while others may feel tense, rejected, or lack a sense of belonging. This difference is consistent with discussions in existing research regarding the heterogeneity of exercise motivation, physical activity experience, and psychological benefits (Wang et al., 2020; Zhong, 2024). To support a more holistic interpretation, recent evidence, such as the DE-PASS systematic review, underscores that the physical activity-mental health nexus cannot be fully understood through physical activity volume alone; it is embedded in a complex web of modifiable individual, social, and environmental determinants across diverse settings (Ciaccioni et al., 2026).

Thus, explaining the cross-sectional association between physical activity and mental well-being solely from the perspective of “exercise volume” may have certain limitations. The psychological benefits associated with physical activities may be related not only to the degree of participation at the behavioral level, but may also be related to an individual’s cognitive evaluation of their own physical abilities, participation motivation, and the emotional support and belonging experience they obtain in the sports environment. Therefore, it is necessary to further investigate the psychological mechanisms in physical activity contexts, especially the potential bridging roles of perceived physical literacy and the sense of belonging in the sports environment in enhancing students’ mental health.

### 1.2 Educational context: a paradigm shift from “physical fitness testing” to inclusive physical education

In the practice of physical education in colleges and universities, physical fitness tests, skills assessments, and sports performance have long been important bases for evaluating students’ sports participation. This evaluation method has the advantages of clear operation and easy management; however, if sports performance and standard-reaching results are overemphasized, it may also overlook students’ subjective experiences during sports participation. For students with weak sports foundations, average physical conditions, or a lack of sports confidence, a single performance orientation may increase their sense of pressure and avoidance tendencies during sports participation, thereby weakening the health promotion function of physical education.

In recent years, China’s school sports and national fitness policies have gradually emphasized the comprehensive educational value of physical education. The “National Fitness Plan (2021–2025)” proposes that the coordinated development of national fitness and school sports should be promoted to encourage students to form lifelong sports awareness and healthy lifestyles (State Council of the People’s Republic of China, 2021). Echoing this, inclusive physical education emphasizes that physical education classrooms should provide opportunities for fair participation, supportive interaction, and positive experiences for students with different physical conditions, ability foundations, and psychological characteristics (Block and Obrusnikova, 2007; Goodwin and Watkinson, 2000). This concept is also consistent with the emphasis on participation opportunities, environmental support, and individual dignity in physical literacy and inclusive education research (Pushkarenko et al., 2021; Whitehead, 2010). For students with differences in physical conditions, athletic abilities, or special health conditions, sports participation opportunities, environmental accessibility, and a supportive atmosphere are particularly important (Úbeda-Colomer et al., 2019).

Therefore, the focus of college physical education should not merely remain on improving physical fitness levels or completing sports tasks, but should also pay attention to students’ psychological experiences in sports contexts. In other words, physical education needs to answer not only the questions of whether students “participate in sports” and whether their “exercise volume meets the standard,” but also whether students feel competent, accepted, and belong in this environment during sports participation.

### 1.3 Problem statement and research significance

Existing research generally suggests a positive association between physical activity and college students’ mental well-being, but explanations for this association still largely remain at the level of behavioral indicators such as exercise frequency, duration, or intensity (Han et al., 2025b; Malagodi et al., 2025; Talapko et al., 2021). Understanding the mental health benefits of physical activity solely from the perspective of “exercise volume” easily neglects individuals’ cognitive evaluations, emotional experiences, and social connections in sports contexts. In fact, even with similar opportunities for sports participation, different students’ subjective experiences may vary significantly: some students can obtain a sense of competence, acceptance, and positive emotions from physical education classes or campus sports activities, while others may feel anxious, alienated, or even rejected. This suggests that the association between physical activity and mental well-being may depend not only on whether or how much one exercises, but also on how individuals understand their own physical abilities and whether they feel a sense of belonging and support in the sports environment.

Perceived physical literacy provides an important perspective for understanding these differences. Physical literacy is usually seen as a comprehensive structure of motivation, confidence, physical competence, knowledge, and understanding required for an individual to pursue lifelong physical activity (Cairney et al., 2019; Whitehead, 2010), and is closely linked to college students’ mental health, resilience, life satisfaction, and psychological adaptation (Kan et al., 2024; Leung et al., 2025; Ma et al., 2021). Higher physical literacy may make students more willing to actively participate in physical activities and form positive self-evaluations in sports contexts. At the same time, the sense of belonging in the sports environment reflects the acceptance, integration, and psychological safety students feel in physical education classes, campus sports, or collective sports activities. Existing research shows that a sense of belonging is an important psychosocial resource for students’ psychological adaptation and well-being (Storey et al., 2024; Yildirim et al., 2021). Therefore, the sense of belonging in the sports environment may represent an important psychological correlate or possible explanatory pathway linking physical literacy and mental well-being.

Based on this, the present study incorporates perceived physical literacy, the sense of belonging in the sports environment, and mental health into the same analytical framework, focusing on examining the predictive role of physical literacy on college students’ mental health, as well as the mediating role of the sense of belonging in the sports environment between the two. Meanwhile, considering that the level of extracurricular physical activity may affect the transformation of sports experiences into mental health benefits, this study further divides exercise groups based on GLTEQ-LSI scores to test whether this moderates the relationship between the sense of belonging in the sports environment and mental health. In addition, this study also examines whether high school sports participation levels and university sports club participation have interactive effects on physical literacy and the sense of belonging in the sports environment.

Specifically, this study aims to answer four questions: First, is perceived physical literacy positively associated with college students’ mental well-being? Second, does the sense of belonging in the sports environment statistically mediate the cross-sectional association between physical literacy and mental well-being? Third, do exercise groups classified based on GLTEQ-LSI moderate the association between the sense of belonging in the sports environment and mental health? Fourth, do high school sports participation levels and university sports club participation have interactive effects on physical literacy and the sense of belonging in the sports environment?

## 2 Theoretical basis and research hypotheses

### 2.1 The foundational link between physical literacy and mental health

As the psychological cornerstone for individuals to engage in physical activities, the theoretical value of perceived physical literacy has been continuously emphasized in physical literacy research (Cairney et al., 2019; Whitehead, 2010). Looking at its structural logic, physical literacy is by no means a simple synonym for athletic ability. The Perceived Physical Literacy Instrument developed by Sum et al. emphasizes that physical literacy not only involves athletic ability but also includes dimensions such as confidence, knowledge and understanding, motivation, and social interaction (Sum et al., 2018). This means that students showing similar performance under the single dimension of physical fitness test scores might differ significantly in the richness of their perceived physical literacy.

Existing studies have found positive relationships between physical literacy and college students’ mental health, psychological resilience, life satisfaction, and positive functional states (Kan et al., 2024; Liu et al., 2025; Ma et al., 2021). The deep logic of this connection is that physical literacy not only provides the technical capital to participate in sports but also shapes a sense of efficacy of “I can do it.” For college students, this confidence derived from the physical level often overflows and reshapes their overall psychological resilience, allowing them to maintain better psychological elasticity amidst heavy academic and interpersonal challenges. This inference is also consistent with research findings that physical activity affects emotional competence and psychological states through self-efficacy (Wang et al., 2020).

*H1*: Perceived physical literacy exhibits a positive structural association with college students’ mental health (WHO-5).

### 2.2 The mediating role of sense of belonging in the sports environment: based on self-determination theory

The term “sense of belonging” is often confused with a simple feeling of liking; however, in the context of physical education, its connotation is far richer than superficial emotional preference. Self-determination theory provides an important theoretical framework for understanding the psychological role of the sense of belonging in the sports environment (Deci and Ryan, 2000). Deci and Ryan (2000) suggested that humans have three basic psychological needs: autonomy, competence, and relatedness, among which the satisfaction of relatedness needs can promote individuals’ intrinsic motivation, positive adaptation, and well-being.

Analyzing the symbiotic relationship between physical literacy and a sense of belonging reveals that students with higher literacy, due to greater confidence, show lower defensive tendencies in collective interactions, making it easier for them to establish deep emotional connections with teachers and peers. This benign interaction satisfies the individual’s social needs, thereby generating positive feedback at the mental level. In the context of physical education, students’ autonomy support, competence experience, and relatedness satisfaction can significantly affect their sports motivation, classroom participation, and psychological adaptation (Standage et al., 2005). This reminds us that the satisfaction of belonging is not just a medium for health benefits; it is itself an indispensable component of mental health. Individuals with high physical literacy complete the leap from physical cognition to psychological tranquility through the establishment of a sense of belonging. This logic is also congruent with research findings that physical education affects college students’ mental health through social support and exercise behavior (Han et al., 2025a; Lei et al., 2025).

*H2*: The sense of belonging in the sports environment plays a significant mediating role between perceived physical literacy and mental health.

### 2.3 The moderating role of extracurricular physical activity levels

Even if individuals achieve a consensus in cognition (literacy) and emotion (sense of belonging), if there is a lack of implementation at the behavioral level, the relational path of health promotion may face the dilemma of insufficient motivation. The frequency and quality of college students’ actual extracurricular sports participation—that is, whether they develop a regular level of extracurricular physical activity—may act as a kind of “power amplification” or “dynamic correction” on the aforementioned paths. Physical activity level is generally considered an important behavioral basis for sports participation affecting mental health, but its role may be influenced collectively by exercise intensity, activity quality, and social context factors (Han et al., 2025b; Malagodi et al., 2025).

According to habit reinforcement theory, regular physical activity not only directly produces physiological gains but also forms a behavioral anchor, allowing the psychological satisfaction brought by the sense of belonging to be continuously reinforced in actual exercise feedback. Relevant studies indicate that exercise behavior and social adaptation may form a chain mediation mechanism between physical activity and college students’ mental health (Han et al., 2025b). For students who are active outside of class, the psychological nourishment they obtain through a sense of belonging on the sports field may translate into a stable mental health index with higher efficiency. Nevertheless, we also observed through data that the health-predictive efficacy of the sense of belonging has a certain robustness; whether it also exerts a similar “psychological protection” role among non-active exercise groups constitutes a deep point of interest for the hypothesis testing here.

*H3*: Extracurricular exercise groups classified based on GLTEQ-LSI play a moderating role between the sense of belonging in the sports environment and mental health; specifically, the positive association between the sense of belonging and mental health is stronger in the active exercise group.

### 2.4 The interactive effect of early sports trajectories and university sports club participation

College students do not enter the university sports field as a “blank slate”; their past sports participation experiences may affect their current physical literacy and sports environment experiences. A higher level of sports participation during high school may continuously influence an individual’s physical literacy upon entering college through the accumulation of sports skills, formation of sports confidence, and peer interaction experiences. Physical literacy theory emphasizes that individual physical activity experiences possess developmental and life-course characteristics, and early experiences may affect subsequent sports confidence, participation motivation, and physical activity behavior (Cairney et al., 2019; Whitehead, 2010). At the same time, university sports clubs, as informal, peer-oriented sports participation spaces, may also provide new socialization opportunities for students with less early sports participation, allowing them to reconstruct sports identity and a sense of belonging in sports during their college years. University belonging and peer support are considered important resources for students’ psychological adaptation, particularly prominent in collective activities and campus communities (Storey et al., 2024; Yildirim et al., 2021).

Therefore, this study further examines whether there is an interactive effect between high school sports participation level and university sports club participation, that is, whether university sports club participation changes the relationship between high school sports participation level and current physical literacy and sense of belonging in the sports environment.

*H4*: High school sports participation level and university sports club participation show an interactive association with college students’ physical literacy and sense of belonging in the sports environment.

### 2.5 Integrated theoretical model and hypotheses

**Figure 1:**
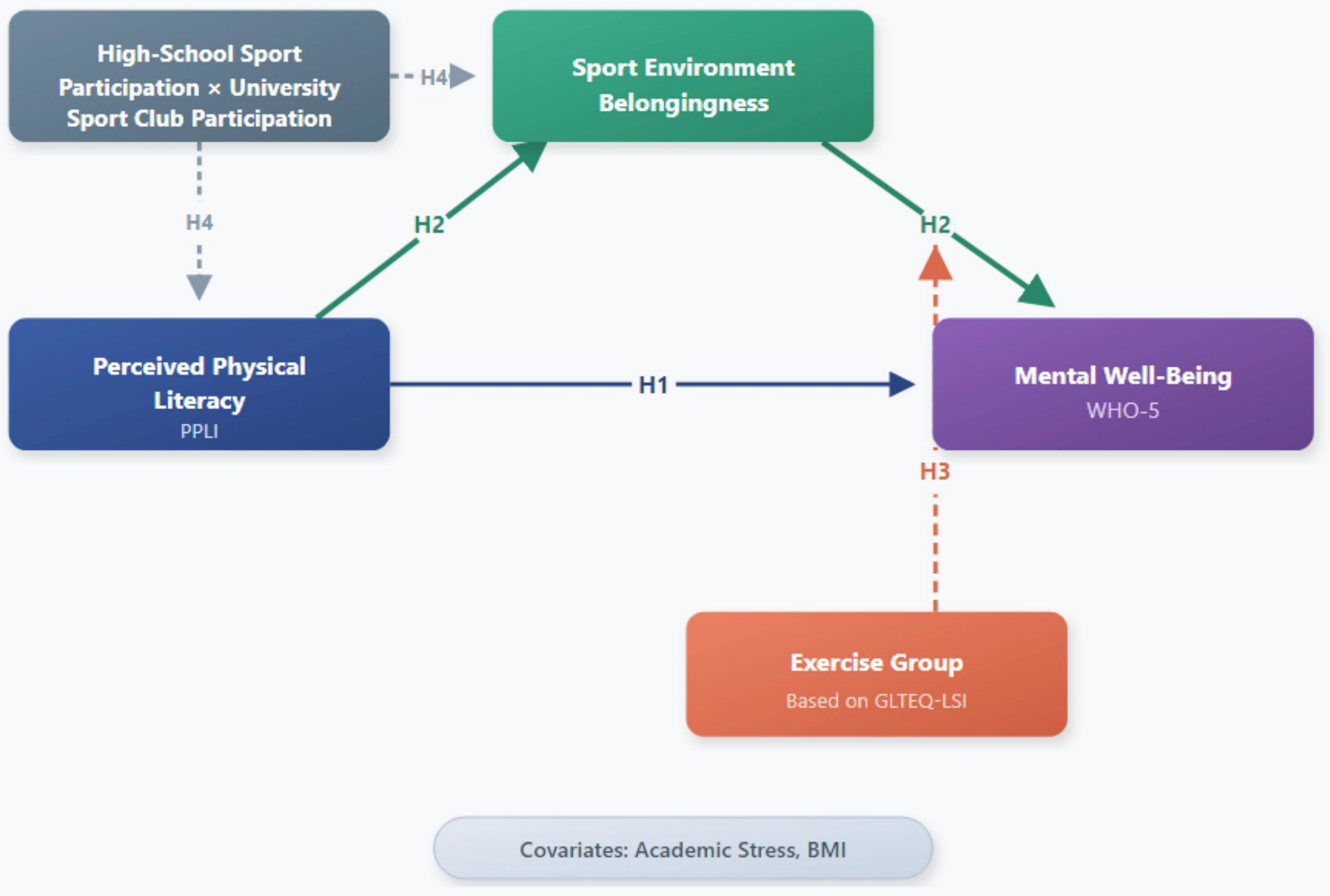
Theoretical model of physical literacy, sense of belonging in the sports environment, and mental health.

To visually present the theoretical hypotheses of this study regarding thecross-sectional relationships among perceived physical literacy, sense of belonging in the sports environment, exercise groups, and mental health, a theoretical research model was constructed as shown in Figure 1.

Figure 1 illustrates the theoretical hypothesis model of this study. Perceived physical literacy is hypothesized to be directly associated with college students’ mental well-being and indirectly associated with mental well-being through the sense of belonging in the sports environment; exercise groups classified based on GLTEQ-LSI are hypothesized to moderate the association between the sense of belonging in the sports environment and mental health; high school sports participation levels and university sports club participation are hypothesized to have an interactive effect on perceived physical literacy and the sense of belonging in the sports environment. The model includes academic stress and BMI as covariates.

## 3 Research methods

### 3.1 Participants and sampling procedure

This cross-sectional study was conducted among college students from three comprehensive universities in central and eastern China. Data collection took place between September and November 2025. This timeframe was specifically selected to capture students’ typical campus routines, avoiding major national holidays that could irregularly disrupt their baseline physical activity patterns.

Prior to data collection, an *a priori* power analysis using G*Power 3.1 indicated that a minimum of 435 participants was required to adequately detect a small-to-medium effect size (*f*^2^ = 0.05) in multiple regression analyses (assuming *α* = 0.05, power = 0.95, and 8 predictors). To ensure robust statistical power after data screening, we set a higher initial recruitment target.

The survey links were distributed through the campus learning management platforms and class communication groups. Students autonomously completed the electronic questionnaires on their personal mobile devices during their free time. The absence of direct physical supervision by researchers during the survey administration was intended to provide a comfortable environment, thereby encouraging candid responses. All participants received an informed consent statement before filling out the questionnaire, understanding that their data would be processed strictly anonymously and used exclusively for academic research, and voluntarily chose whether to participate.

The original collected data underwent two rounds of systematic cleaning: The first round used health screening items in the questionnaire to exclude participants who self-reported long-term physical injuries or physical limitations and were currently unable to participate in daily physical activities autonomously; the second round calculated Z-scores for three core continuous variables—physical literacy (PPLI), sense of belonging, and WHO-5—and verified and deleted extreme values exceeding ±3.0. The original number of returned questionnaires was 950. After cleaning, 890 valid questionnaires were retained, and 60 invalid questionnaires were excluded, yielding a valid response rate of 93.68%. The cleaned data had no missing values, and all core items entered subsequent statistical analysis.

The final valid sample size included in the statistical analysis was 890, comprising 465 males (52.2%) and 425 females (47.8%). The participants’ ages ranged from 18 to 23 years, with a mean age of 20.48 years (*SD* = 1.70). The average BMI of the participants was 21.44 (*SD* = 2.76), falling within the normal weight range; the mean academic stress score was 2.98 (*SD* = 1.42), indicating that the perceived academic burden of the sample as a whole was at a moderate level. In addition, the sample was relatively balanced in terms of grade, major category, place of origin, family socioeconomic status, and physical education class participation. Details are shown in Table 1.

**Table 1:** Basic demographic characteristics of the sample.

Characteristic	Category/statistic	Number/value	Percentage/SD
Gender	Male	465	52.2%
	Female	425	47.8%
Age	Mean ± SD	20.48	±1.70
Age range	Min–Max	18–23	—
BMI	Mean ± SD	21.44	±2.76
Academic stress	Mean ± SD	2.98	±1.42
Grade	Freshman	259	29.1%
	Sophomore	269	30.2%
	Junior	226	25.4%
	Senior and above	136	15.3%
Major category	Humanities/Social Sciences/Management	209	23.5%
	Science/Engineering/Agriculture/Medicine	211	23.7%
	Arts/Media	224	25.2%
	Sports-related majors	246	27.6%
Place of origin	Urban (including county towns)	444	49.9%
	Rural	446	50.1%
Subjective family economic status	Poor/Below average	123	13.8%
	Moderate/Average	634	71.2%
	Good/Above average	133	14.9%
High school sports participation	Hardly participated in sports/Skipped PE due to studies	230	25.8%
	Attended normal PE classes, occasionally exercised extracurricularly	210	23.6%
	Frequently exercised extracurricularly but had no systematic training	214	24.0%
	Was a member of school teams or received high-intensity systematic training	236	26.5%
University sports club participation	Participated	459	51.6%
	Did not participate	431	48.4%
PE course enrollment this semester	Currently enrolled in PE class	472	53.0%
	Not currently enrolled in PE class	418	47.0%
Exercise level, based on LSI	Active group (LSI ≥ 24)	737	82.8%
	Moderately active group (LSI 14–23)	133	14.9%
	Insufficiently active group (LSI < 14)	20	2.2%

### 3.2 Measurement instruments

The questionnaire used in this study consisted of six parts: research instructions and informed consent, demographic and baseline characteristics, the GLTEQ leisure-time physical activity questionnaire, the perceived physical literacy instrument, the sense of belonging in the sports environment scale, and the WHO-5 subjective well-being index. The complete questionnaire is available in Supplementary material 1, and the variable codebook is in Supplementary material 2.

#### 3.2.1 Leisure-time physical activity questionnaire (GLTEQ) and exercise group definition

This study used the Godin-Shephard Leisure-Time Physical Activity Questionnaire to assess the frequency of participants engaging in high, moderate, and mild intensity leisure sports activities during a typical week (Godin and Shephard, 1985; Godin, 2011). Weight coefficients were determined based on metabolic equivalent conversions: high-intensity activities (sweating heavily, rapid heartbeat, e.g., fast running, HIIT) have a coefficient of 9; moderate-intensity activities (light sweating, e.g., brisk walking, recreational ball games) have a coefficient of 5; mild-intensity activities (little change in heart rate, e.g., walking, yoga) have a coefficient of 3. These were used to calculate the Leisure Score Index (LSI) as shown in Equation 1:


$$\mathrm{LSI}=9\times f_{\text{high}-\text{intensity}}+5\times f_{\text{moderate}}+3\times f_{\text{mild}}$$ (1)

While utilizing continuous metabolic scores can offer granular data, categorizing physical activity levels aligns with public health guidelines for differentiating “active” versus “insufficiently active” populations, which facilitates the formulation of practical health promotion strategies. According to Godin’s (2011) standard cutoff values, those with an LSI ≥ 24 were classified into the active exercise group, those with 14 ≤ LSI < 24 into the moderately active group, and those below 14 into the insufficiently active group. In subsequent difference tests and moderation effect analyses, the moderately active group and insufficiently active group were merged into a control group (*n* = 153) to ensure comparability of sample sizes between groups.

Furthermore, to address the potential overestimation of physical activity levels caused by the inclusion of mild activities in the traditional LSI, a Health Contribution Score (HCS) was calculated according to Godin’s updated recommendations (Godin, 2011). The HCS focuses strictly on moderate-to-vigorous physical activity (MVPA) by excluding mild-intensity items (Equation 2):


$$\mathrm{HCS}=9\times f_{\text{high}-\text{intensity}}+5\times f_{\text{moderate}}$$ (2)

The HCS was primarily utilized as a sensitivity reference to verify the robustness of the exercise group classification.

#### 3.2.2 Perceived Physical Literacy Instrument (PPLI)

The independent variable used the Perceived Physical Literacy Instrument (PPLI) developed by Sum et al. (2018), with wording adjustments tailored to the context of this study. During its validation among Chinese cohorts, the instrument demonstrated strong construct validity and acceptable internal reliability, yielding Cronbach’s *α* values between 0.73 and 0.81. It consists of 5 items covering aspects such as sports confidence, health cognition, peer collaboration, sports knowledge, and intrinsic motivation, scored on a 5-point Likert scale (1 = *strongly disagree*, 5 = *strongly agree*). This study took the mean of the 5 items as the total PPLI score. A higher score indicates a higher level of perceived physical literacy for the participant. The Cronbach’s α of the scale in this study was 0.778, indicating good internal consistency reliability.

#### 3.2.3 Sense of belonging in the sports environment scale

The mediating variable was measured using a self-developed Sense of Belonging in the Sports Environment Scale. The initial item pool was generated based on the relatedness dimension of Self-Determination Theory (Deci and Ryan, 2000) and existing literature on school belongingness. To establish content validity, a panel of three academic experts in sports psychology evaluated the constructs, resulting in minor wording refinements tailored to the college physical education context. Subsequently, a pilot test (*n* = 50) was conducted to confirm item clarity and preliminary internal consistency. The final scale comprising 5 items that focus on assessing the degree of peer acceptance, teacher support, group integration, and psychological safety felt by the participants in PE classes and campus group sports activities, such as “During PE classes or campus sports activities, I feel completely integrated into this group.” The scale used a 5-point Likert scale (1 = strongly disagree, 5 = strongly agree). The mean of the 5 items was used as the score for the sense of belonging in the sports environment, with higher scores representing a stronger sense of belonging in the sports environment. The *α* in this sample was 0.741.

#### 3.2.4 World Health Organization five Well-Being Index (WHO-5)

The dependent variable used the WHO-5 Well-Being Index, a scale widely employed to assess individuals’ recent positive emotions, vitality, and subjective well-being, which has consistently demonstrated high sensitivity and acceptable internal consistency (Cronbach’s α > 0.80) across diverse global populations (Topp et al., 2015). It includes 5 items measuring the participants’ positive mood, vitality, and daily functional state over the past 2 weeks, such as “I have felt cheerful and in good spirits.” This study used a 0–5 scoring method (0 = *at no time*, 5 = *all of the time*), taking the mean of the 5 items as the WHO-5 score. Higher scores indicate a more positive recent subjective physical and mental adaptation state. The WHO-5 has robust reliability and validity in cross-cultural contexts. In this study, Cronbach’s α was 0.780. “Mental health” in this paper mainly refers to the positive emotions, vitality, and subjective well-being state reflected by the WHO-5, and is not equivalent to a clinical diagnosis of psychological disorders.

#### 3.2.5 Control variables

Based on existing literature and considering sample characteristics, this study included academic stress and BMI as control variables to exclude their potential confounding effects on the core paths. Academic stress was measured using a single-item 5-point Likert scale (1 = *very low*, 5 = *very high*); BMI was calculated based on participants’ self-reported height and weight using Equation 3:


$$\mathrm{BMI}=\frac{\text{Weight}(\mathrm{kg})}{\text{Height}{\left(m\right)}^{2}}$$ (3)

Additionally, gender, age, grade, and family economic status were included in the extended model’s collinearity diagnostics.

### 3.3 Data analysis strategy

All statistical analyses were completed in the Python 3.11 environment (pandas, scipy, statsmodels, scikit-learn, semopy), and cross-verified referring to the SPSS 26.0 analysis logic. Before formal statistics, data completeness, missing values, outliers, and the distribution of core variables were first checked. No missing values were found in the cleaned data, so no imputation was performed. The BMI, total LSI score, HCS moderate-to-vigorous physical activity score, as well as the mean scores of the three core scales (PPLI, belongingness, and WHO-5) were then calculated.

Categorical variables were reported with actual category names in descriptive statistics. In regression analysis, nominal categorical variables were generally treated as dummy variables specifying reference groups; ordinal categorical variables were treated as ordinal based on their rank order.

1. Common method bias testing used Harman’s single-factor test, incorporating all 15 scale items into a principal component analysis to evaluate whether the variance explained by a single factor was too high (Podsakoff et al., 2003).
2. Scale reliability and validity testing: Cronbach’s *α*, Confirmatory Factor Analysis (CFA) using the Maximum Likelihood (ML) estimator, standardized factor loadings, Composite Reliability (CR), Average Variance Extracted (AVE), and the fit comparison between the three-factor model and the single-factor model;
3. Descriptive statistics and normality testing: Mean, standard deviation, minimum, maximum, skewness, kurtosis, and K-S test;
4. GLTEQ specific analysis: Frequency characteristics of activities at each intensity, LSI group distribution, and gender difference t-tests;
5. Sensitivity analysis to compare the differences in exercise classifications when using LSI (including mild activities) versus HCS (excluding mild activities);
6. Between-group difference comparisons: Independent samples t-tests and *χ*^2^ tests;
7. Regression assumptions testing: Assessing residual normality, linearity, homoscedasticity, and independence (Durbin-Watson test), as well as detecting influential observations or outliers (e.g., Cook’s distance) prior to path modeling;
8. H1 direct path: OLS regression to test the direct predictive effect of physical literacy on mental health;
9. H2 mediating effect: Hierarchical OLS regression + Bootstrap resampling estimation of the 95% confidence intervals for indirect effects;
10. H3 moderating effect: Incorporating the interaction term of belongingness × exercise group to test whether the exercise group moderates the association between belongingness and mental health;
11. H4 interactive effect: Two-way ANOVA to test the impact of high school sports participation level × university sports club participation on physical literacy and belongingness;
12. Model diagnostics: Calculating VIF indicators in extended models including major predictive variables and demographic variables to test multi-collinearity risks.

## 4 Results

### 4.1 Common method bias test

Since the data for this study stem from self-report questionnaires completed at the same time point, common method bias needs to be systematically evaluated. All 15 items of the PPLI, belongingness, and WHO-5 scales were incorporated into a principal component analysis. The results showed: Overall KMO for the 15 items = 0.889, Bartlett’s test of sphericity was significant, *χ*^2^ = 3300.66, *df* = 105, *p* < 0.001, indicating that the data were suitable for factor structure testing. The variance explained by the first unrotated principal component was 30.05%, below the common threshold, meaning there was no obvious dominant single-method factor issue (Podsakoff et al., 2003). The explanatory rates of the first three principal components were 30.05, 12.53, and 9.45% respectively, and no single factor occupied an absolute dominant position. These results indicate that there is no serious common method bias problem in the data, providing appropriate methodological guarantees for subsequent statistical results.

### 4.2 Reliability and validity test of the measurement model

The internal consistency, CFA standardized loadings, composite reliability, and AVE results of the three core scales are shown in Table 2. The results show that the Cronbach’s *α* for PPLI, sense of belonging in the sports environment, and WHO-5 are 0.778, 0.741, and 0.780, respectively, all reaching the general standard of above 0.70, indicating that the scales have acceptable internal consistency.

**Table 2:** Results of the three-factor CFA measurement model.

Latent variable	Number of items	Cronbach’s *α*	Standardized loadings range	CR	AVE
PPLI physical literacy	5	0.778	0.629–0.666	0.778	0.412
Sense of belonging in sports environment	5	0.741	0.585–0.630	0.741	0.365
WHO-5 mental health	5	0.780	0.599–0.689	0.781	0.418

This study further employed Confirmatory Factor Analysis (CFA) to test the three-factor measurement model and the single-factor alternative model; fit index comparisons are presented in Table 3. The results showed that all fit indices of the three-factor model met or exceeded the judgment standards (*χ*^2^/*df* = 0.989, CFI = 1.000, TLI = 1.000, RMSEA < 0.001), and were significantly better than the single-factor model structure, indicating the three-factor model has good goodness-of-fit. This supports physical literacy, sense of belonging in the sports environment, and WHO-5 mental health as mutually independent yet interconnected constructs, which aligns with the understanding of construct distinctiveness in existing studies using the PPLI and WHO-5 scales (Sum et al., 2018; Topp et al., 2015).

**Table 3:** Comparison of fit indices between the three-factor model and single-factor model.

Model	χ2	df	χ2/df	*p*	CFI	TLI	RMSEA	GFI	AGFI	NFI
Three-factor model	86.012	87	0.989	0.510	1.000	1.000	0.000	0.974	0.969	0.974
Single-factor model	1066.361	90	11.848	< 0.001	0.697	0.646	0.110	0.679	0.626	0.679

In terms of standardized factor loadings, the items in the three-factor model ranged from 0.585–0.689, and all estimates were significant. The composite reliability (CR) ranged from 0.741–0.781, meeting standards. The Average Variance Extracted (AVE) ranged from 0.365–0.418. Although these values fall below the ideal 0.50 threshold, internal consistency is still considered adequate because the CR values well exceeded the recommended 0.70 benchmark. According to Fornell and Larcker (1981), if the CR is acceptable, the convergent validity of the constructs is generally considered sufficient for continuing structural analysis, though interpretations should be made with appropriate academic caution. Overall, CFA results supported the global structure of the three-factor measurement model in this study.

### 4.3 Descriptive statistics and correlation analysis of variables

The descriptive statistics and normality test results of core variables are shown in Table 4, and the Pearson correlation coefficient matrix is in Table 5.

**Table 4:** Descriptive statistics and normality tests of core variables.

Variable	M	SD	Min	Max	Skewness	Kurtosis	K-S p
Age	20.483	1.702	18.00	23.00	0.015	−1.264	< 0.001
BMI	21.444	2.761	16.33	32.04	0.252	−0.192	0.305
Academic stress	2.979	1.421	1.00	5.00	0.005	−1.305	< 0.001
Physical literacy (PPLI)	3.000	0.493	1.40	4.80	0.028	0.030	< 0.001
Sense of belonging in sports environment	2.857	0.475	1.60	4.40	0.065	−0.116	< 0.001
Mental health (WHO-5)	2.478	0.593	0.80	4.20	0.031	−0.029	< 0.001
High-intensity activity frequency	1.573	1.278	0.00	6.00	0.688	0.093	< 0.001
Moderate-intensity activity frequency	2.498	1.610	0.00	9.00	0.609	0.469	< 0.001
Mild-intensity activity frequency	4.030	2.048	0.00	12.00	0.465	0.224	< 0.001
Total LSI score	38.737	15.823	6.00	91.00	0.536	0.083	0.007
HCS moderate-vigorous score	26.646	14.307	0.00	76.00	0.558	0.214	< 0.001

K-S = Kolmogorov–Smirnov test for normality. Because of the large sample size, the K-S test is sensitive to minor deviations. The absolute values for skewness and kurtosis of core continuous variables were all low, indicating that the overall deviation of variables from a normal distribution was limited.

**Table 5:** Pearson correlation coefficient matrix of core variables.

Variable	1	2	3	4	5	6	7	8
1. Physical literacy (PPLI)	—							
2. Sense of belonging in sports environment	0.396***	—						
3. Mental health (WHO-5)	0.299***	0.416***	—					
4. Academic stress	−0.022	0.010	−0.004	—				
5. BMI	0.027	−0.005	−0.001	0.010	—			
6. Age	−0.013	−0.008	0.006	−0.014	−0.037	—		
7. Total LSI score	0.014	−0.018	−0.061	−0.020	0.001	0.008	—	
8. HCS moderate-vigorous score	0.017	0.003	−0.050	−0.007	0.017	0.001	0.922***	—

***p < 0.001; *n* = 890.

The correlation analysis revealed a clear relational structure: There was a moderate positive correlation between physical literacy and sense of belonging (*r* = 0.396, *p* < 0.001), the correlation between physical literacy and mental health was relatively weaker but significant (*r* = 0.299, *p* < 0.001), and the correlation between the sense of belonging and mental health was the strongest (*r* = 0.416, *p* < 0.001). This pattern preliminarily suggests that the sense of belonging may play an important mediating role between physical literacy and mental health. Furthermore, the correlation coefficients between academic stress, BMI, and the three core variables did not exceed 0.03, indicating weak linear correlations. However, they were still controlled in subsequent models to improve the robustness of the path estimates. Notably, total LSI scores and HCS scores had weak correlations with WHO-5, indicating that simple physical activity volume metrics cannot adequately explain the mental health level of college students. In contrast, the sense of belonging in the sports environment had the highest correlation with WHO-5, further supporting its potential key bridging role between physical literacy and mental health.

### 4.4 Direct association between physical literacy and mental well-being (H1)

Prior to running the regression, preliminary checks confirmed that the assumptions of residual normality, linearity, and homoscedasticity were met, and no influential outliers were detected (maximum Cook’s distance < 0.1). After controlling for academic stress and BMI, OLS regression was performed with physical literacy (PPLI) as the independent variable and mental health (WHO-5) as the dependent variable. The results are shown in Table 6.

**Table 6:** OLS regression results predicting mental well-being outcomes from physical literacy.

Predictor	*b* (Unstandardized)	*SE*	*t*	*p*	95% CI
Physical literacy (PPLI)	0.360	0.039	9.32	< 0.001	0.284,0.435
Academic stress	0.001	0.013	0.08	0.934	−0.025,0.027
BMI	−0.002	0.007	−0.29	0.775	−0.015,0.012
Constant	1.439	0.190	7.56	< 0.001	1.065,1.812

R^2^ = 0.089, adj-R^2^ = 0.086, F (3, 886) = 28.99, *p* < 0.001.

Physical literacy had a significant positive explanatory effect on mental health (*β* = 0.299, *p* < 0.001). The model explained approximately 8.9% of the variance, representing a small-to-medium effect size, which is common in cross-sectional surveys in the social sciences. Neither academic stress nor BMI showed significant predictive power for WHO-5, after controlling for them, the positive predictive effect of physical literacy remained robust. H1 is statistically supported.

### 4.5 Specialized analysis of GLTEQ leisure-time physical activity behavior

#### 4.5.1 Descriptive characteristics of extracurricular physical activities and LSI distribution

**Figure 2:**
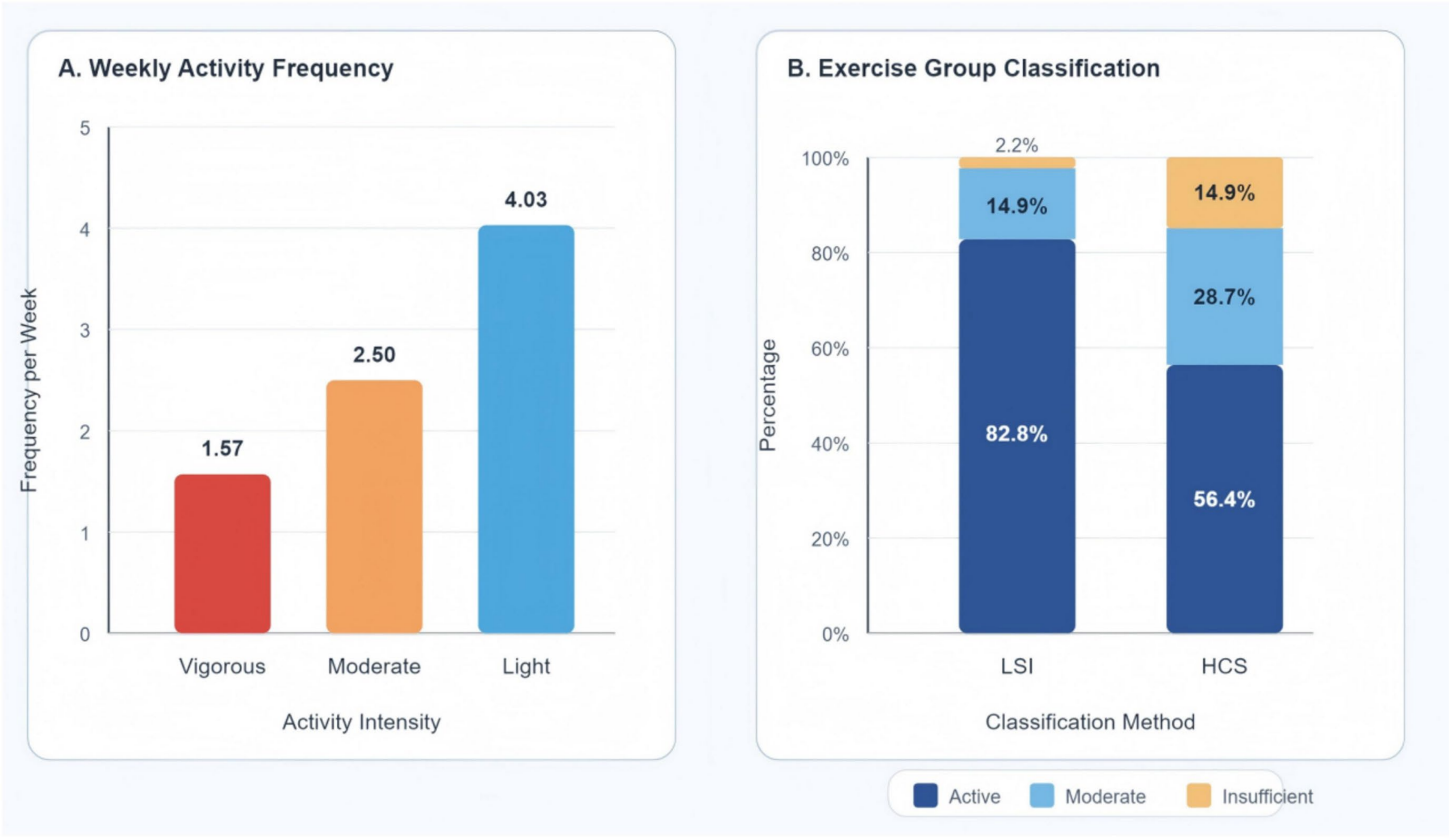
GLTEQ physical activity structure and LSI/HCS grouping comparison. Chart **(A)** shows the average weekly frequencies of extracurricular physical activities of different intensities; Chart **(B)** shows the distribution of exercise classes based on LSI and HCS standards. LSI includes high, moderate, and mild-intensity activities, whereas HCS includes only high and moderate-intensity activities.

The descriptive distribution of physical activities at different intensities, along with their corresponding classification categories, is visually summarized in Figure 2A. Notably, none of the participants reported a total LSI score of zero (Min = 6), indicating that all students engaged in at least a baseline level of mild-intensity movement. As illustrated in Figure 2A, the three intensities of activity showed a clear “inverted triangle” distribution: as exercise intensity increased, the frequency of participation decreased significantly. Mild-intensity activities had the highest average weekly frequency (*M* = 4.03), dominated by low-load activities like walking and light stretching; moderate-intensity activities were next (*M* = 2.50); high-intensity activities were the lowest (*M* = 1.57), suggesting that the extracurricular exercise structure of college students tilts toward mild levels, leaving significant room to improve high-intensity exercise participation.

According to Godin’s (2011) standard cutoff values, the three-tier distribution was as follows: The active exercise group (LSI ≥ 24) accounted for 82.8% (737 students), the moderately active group (14 ≤ LSI < 24) accounted for 14.9% (133 students), and the insufficiently active group (LSI < 14) accounted for 2.2% (20 students). In absolute proportions, over 80% of the students met the health exercise threshold; however, it is worth noting that within its score composition, mild-intensity activities contributed about 31% (mean 4.03 × 3 = 12.09 points), and high-intensity activities contributed about 36% (mean 1.57 × 9 = 14.13 points), reflecting an exercise ecology of “meeting standards but with low quality.” Looking at the score breakdown, mild-intensity activities contributed heavily to the total LSI score, suggesting that using the LSI indicator that includes mild-intensity activities might inflate the proportion of the active exercise group. To further test this issue, this study added a sensitivity analysis of HCS moderate-to-vigorous physical activity scores in the next section.

#### 4.5.2 Sensitivity analysis of HCS moderate-vigorous physical activity score

Considering that health contributions are primarily driven by moderate-to-vigorous physical activity, this study conducted a sensitivity analysis using the HCS. Compared to the total LSI, which includes mild-intensity activities, the HCS explicitly removes their contributions to reassess the actual prevalence of active exercise. The results showed that when grouped using the total LSI score, the active exercise group accounted for 82.8%; but when grouped using the HCS moderate-to-vigorous physical activity score, the active exercise group proportion fell to 56.4%. Simultaneously, the insufficiently active group proportion rose from 2.2 to 14.9%. This difference indicates that including mild-intensity activities in the LSI score may inflate the active exercise group ratio. Therefore, this study remained cautious when interpreting the results of the exercise group and used the HCS as a sensitivity reference indicator for exercise grouping. The specific numbers and percentages of the two grouping methods are shown in Table 7.

**Table 7:** Comparison of LSI and HCS exercise grouping methods.

Grouping standard	Active group	Moderately active group	Insufficiently active group
LSI total score: 9 High + 5 Moderate + 3 Mild	737 (82.8%)	133 (14.9%)	20 (2.2%)
HCS: 9 High + 5 Moderate	502 (56.4%)	255 (28.7%)	133 (14.9%)

The comparative distribution profiles under both classification standards are visually detailed in Figure 2B.

### 4.6 Between-group difference comparisons

#### 4.6.1 Preliminary difference test of exercise groups on the three core variables

Defining LSI ≥ 24 as the active exercise group (*n* = 737) and less than 24 as the control group (*n* = 153), independent samples *t*-tests were conducted for the three core variables, and the results are shown in Table 8.

**Table 8:** Independent samples T-test results for core variables by exercise group.

Variable	Active group *M* ± *SD*	Control group *M* ± *SD*	*t*	*p*	Cohen’s *d*
Physical literacy (PPLI)	2.999 ± 0.490	3.001 ± 0.505	−0.042	0.966	−0.004
Sense of belonging in sports environment	2.849 ± 0.472	2.897 ± 0.488	−1.142	0.254	−0.102
Mental health (WHO-5)	2.483 ± 0.589	2.455 ± 0.612	0.539	0.590	0.048

Cohen’s d < 0.20 indicates a trivial effect; all comparisons are non-significant (*p* > 0.05).

None of the three t-tests reached a significant level, and the absolute values of effect sizes did not exceed 0.11, which falls into a trivial magnitude. This means that, in the context of this study’s sample, the exercise groups defined solely by LSI could not systematically differentiate students in terms of physical literacy, sense of belonging, or mental health. This further suggests that the total volume of physical activity is not necessarily equal to the quality of sports participation—a simple measure of physical activity may not capture all mechanisms affecting psychological states; the underlying cognitive and emotional pathways are the key. These results provide a preliminary background for further interaction testing of H3, but do not alone constitute a judgment on the moderating effect.

#### 4.6.2 Interactive effects of high school sports participation level and university sports club participation (H4)

To test H4, this study re-dichotomized the high school sports participation levels. Participants who selected “Hardly participated in sports/Skipped PE due to studies” or “Attended normal PE classes, occasionally exercised extracurricularly” were grouped into the lower high school sports participation group; those who selected “Frequently exercised extracurricularly but had no systematic training” or “Was a member of school teams or received high-intensity systematic training” (the latter comprising 236 students, or 26.5% of the sample, highlighting a specifically highly active subset within this cultural context) were categorized into the higher high school sports participation group. University sports club participation was divided into participating (*n* = 459, 51.6%) and non-participating (*n* = 431, 48.4%) groups based on Q13. A 2 × 2 two-way ANOVA (Type II SS) of “high school sports participation level × university sports club participation” was constructed with physical literacy (PPLI) and sense of belonging in the sports environment as dependent variables, respectively. The group means are shown in Table 9, and the ANOVA results in Table 10.

**Table 9:** 2 × 2 Group means (*M* ± *SD*).

High school experience	University sports club participation	*n*	PPLI physical literacy	Sense of belonging in sports environment
Lower High school sports participation group	Did not participate	217	2.986 ± 0.521	2.857 ± 0.483
Lower high school sports participation group	Participated	223	2.988 ± 0.503	2.823 ± 0.449
Higher high school sports participation group	Did not participate	214	3.011 ± 0.501	2.906 ± 0.481
Higher high school sports participation group	Participated	236	3.013 ± 0.450	2.844 ± 0.485

The lower high school sports participation group is combined from Q12 options 1–2; the higher group is from Q12 options 3–4. See Supplementary material 1 for specific option contents.

**Table 10:** Two-way ANOVA summary (Type II SS).

Dependent variable	Source of variance	*F*	*p*	Partial *η*^2^
Physical literacy (PPLI)	High school sports participation level (A)	0.557	0.456	0.001
	University sports club participation (B)	0.003	0.956	0.000
	A × B	0.000	0.992	0.000
Sense of belonging in sports environment	High school sports participation level (A)	1.152	0.283	0.001
	University sports club participation (B)	2.254	0.134	0.003
	A × B	0.189	0.664	0.000

All effects are non-significant (*p* > 0.05); Partial η^2^ ≈ 0.000–0.003 indicates a trivial effect.

**Figure 3:**
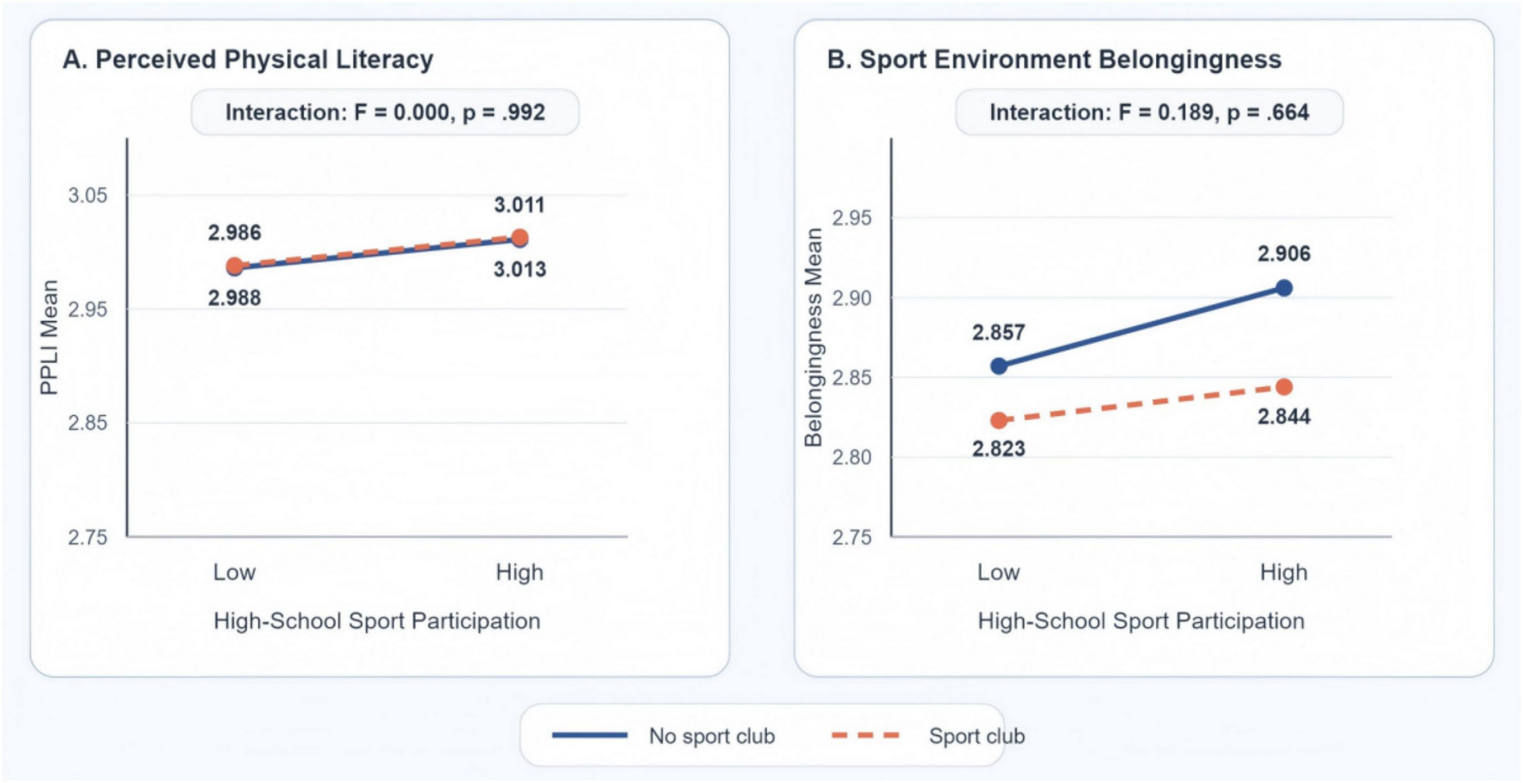
Interaction plots of high school sports participation level and university sports club participation on physical literacy and sense of belonging in the sports environment. Chart **(A)** shows the mean physical literacy scores under different high school sports participation levels and university sports club participation states; Chart **(B)** shows the mean sense of belonging in the sports environment under the same groupings. No sport club means not participating in university sports clubs; Sport club means participating. The interaction effects on both dependent variables did not reach statistical significance, indicating no significant interaction between high school sports participation levels and university sports club participation.

To further visually display the interactive patterns of high school sports participation levels and university sports club participation on physical literacy and belongingness, interaction plots were drawn, as seen in Figure 3.

Two-way ANOVA results showed that whether taking physical literacy or sense of belonging in the sports environment as the dependent variable, high school sports participation levels, university sports club participation, and their interaction terms did not reach significant levels (all *p* > 0.05, partial *η*^2^ < 0.003). The mean differences among the four groups were extremely small, indicating that under the current sample and measurements, high school sports participation levels and university club participation status could not significantly distinguish students’ physical literacy and sense of belonging in the sports environment. H4 was not statistically supported.

### 4.7 Relational path test of physical literacy, sense of belonging, and mental health (H2 & H3)

Based on controlling for academic stress and BMI, OLS hierarchical regression accompanied by the Bootstrap method (5,000 resamples) was used to systematically test the mediation path: physical literacy → sense of belonging → mental health, as well as the moderating role of exercise groups on the relationship between sense of belonging and mental health. The complete path coefficients are shown in Table 11.

**Table 11:** Summary of mediation and moderation path regression coefficients.

Path/effect	*b* (Unstandardized)	*SE*	*t*	*p*	95% CI
Path a: Physical literacy → Belongingness	0.382	0.030	12.85	< 0.001	0.324,0.441
Path b: Belongingness → WHO-5	0.441	0.041	10.75	< 0.001	0.360,0.522
Direct Effect c’: Physical literacy → WHO-5	0.191	0.040	4.84	< 0.001	0.113,0.269
Total Effect c: Physical literacy → WHO-5	0.360	0.039	9.32	< 0.001	0.284,0.435
Indirect effect (Bootstrap)	0.169	—	—	—	0.131,0.209
Moderation term: Belongingness × Exercise group	0.013	0.102	0.13	0.900	−0.187,0.213

Bootstrap 5,000 resamples. A 95% CI excluding 0 indicates a statistically significant indirect effect; Exercise group: 1 = Active, 0 = Control.

**Figure 4:**
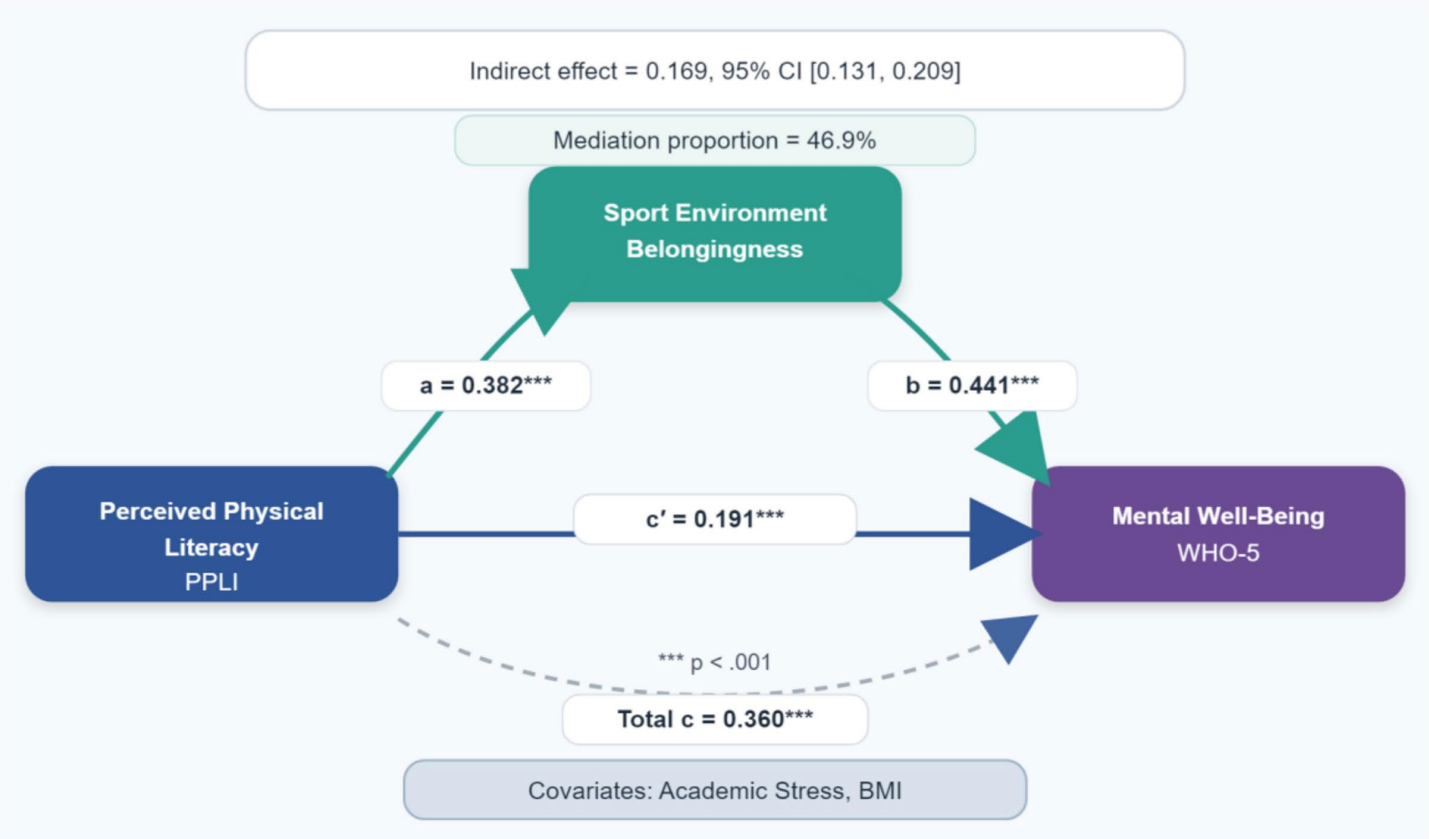
Path diagram of the mediation effect of sense of belonging in the sports environment between physical literacy and mental health. Path coefficients are unstandardized regression coefficients. The model controls for academic stress and BMI. Indirect effects were estimated using Bootstrap with 5,000 resamples. ***p < 0.001.

To present the mediating role of sense of belonging in the sports environment between perceived physical literacy and mental health more intuitively, a path diagram of the mediation effect is shown in Figure 4.

The path analysis results showed that physical literacy could significantly and positively explain variance in the sense of belonging in the sports environment (*a* = 0.382, *β* = 0.396, *p* < 0.001), and similarly, the sense of belonging in the sports environment further significantly and positively correlated with mental health (*b* = 0.441, *β* = 0.353, *p* < 0.001). After incorporating the sense of belonging in the sports environment, the direct effect of physical literacy on mental health remained significant (*c*′ = 0.191, *β* = 0.159, *p* < 0.001), but was lower than the total effect (*c* = 0.360, *β* = 0.299, *p* < 0.001), indicating that the sense of belonging in the sports environment exerted a partial mediating effect between the two. The Bootstrap results showed an indirect effect of 0.169, 95% CI 0.131, 0.209, with the confidence interval excluding 0, supporting H2.

In contrast, the interaction term of belongingness × exercise group was not significant (*b* = 0.013, *p* = 0.900), indicating that exercise groups partitioned based on GLTEQ-LSI did not significantly moderate the association between sense of belonging in the sports environment and mental health. H3 was not statistically supported.

**Figure 5:**
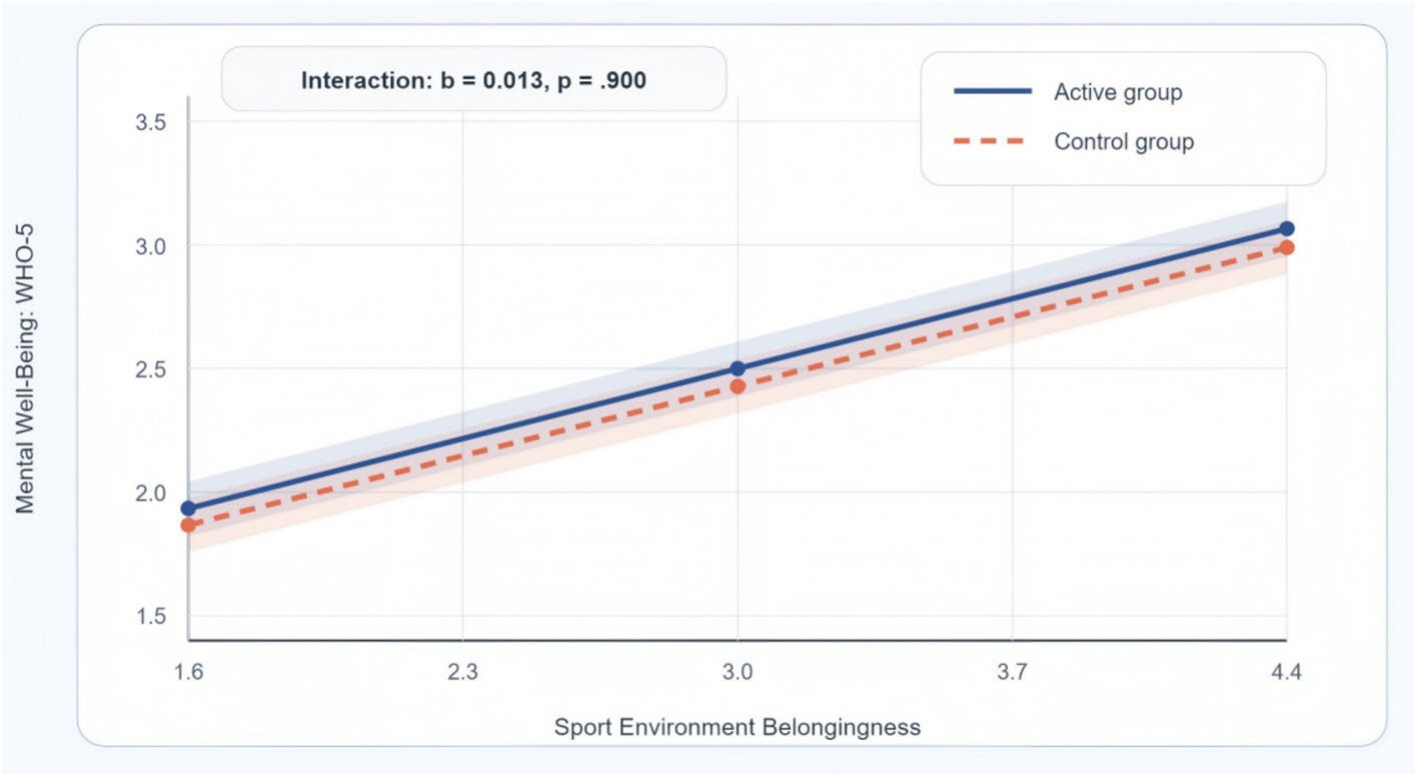
The moderating effect of exercise groups on the relationship between sense of belonging in the sports environment and mental health. The lines in the figure represent the simple slopes was positively associated with by the model. Active group and Control group are based on GLTEQ-LSI groupings. The interaction term was not significant (*b* = 0.013, *p* = 0.900), indicating that the exercise group did not significantly moderate the relationship between the sense of belonging in the sports environment and WHO-5.

To visually display the moderating mode of exercise groups on the relationship between sense of belonging in the sports environment and mental health, a simple slope plot was drawn, as seen in Figure 5.

Figure 5 shows that both regression lines present a positive trend and are approximately parallel, indicating that the positive association between sense of belonging in the sports environment and mental health is relatively stable across different exercise groups.

### 4.8 Regression model diagnostics

To check if the regression model suffered from severe multicollinearity, this study computed the Variance Inflation Factor (VIF) for the main predictors. As shown in Table 12, in the model encompassing physical literacy, sense of belonging in the sports environment, academic stress, BMI, gender, age, grade, and family economic status, the VIF values for all variables ranged from 1.004–1.191, well below the usual threshold of 5, indicating no severe multicollinearity issues in the model.

**Table 12:** Multicollinearity diagnostics of the main regression model.

Variable	VIF
Physical literacy (PPLI)	1.189
Sense of belonging in sports environment	1.191
Academic stress	1.005
BMI	1.005
Gender	1.004
Age	1.004
Grade	1.006
Family economic status	1.004

## 5 Discussion

### 5.1 Explaining the association between physical literacy and mental well-being: the statistical mediating role of belongingness

In the relational framework of this study, perceived physical literacy showed a significant positive association with mental well-being (*b* = 0.360, *p* < 0.001), supporting H1. Physical literacy, as a composite construct encompassing sports confidence, sports knowledge, and participation motivation, reflects an individual’s subjective evaluation of their own athletic ability and the value of sports. This understanding is consistent with the comprehensive definition of motivation, confidence, physical competence, knowledge, and understanding in physical literacy theory (Cairney et al., 2019; Whitehead, 2010). Crucially, university physical education and campus sports environments represent a key developmental transition. For college students—who are in the critical phase of late adolescence and emerging adulthood—these environments serve as the direct continuation and culmination of K-12 school physical education. Understanding how sports-related psychological resources function during this transition is vital for sustaining the physical activity habits established in earlier childhood and youth. The results showed that students with higher physical literacy levels tend to have more positive subjective physical and mental adaptation states. This finding aligns with existing studies reporting positive associations between physical literacy and mental well-being, resilience, life satisfaction, and positive psychological functioning among college students (Kan et al., 2024; Leung et al., 2025; Liu et al., 2025; Ma et al., 2021). This finding suggests that physical literacy is not merely reflected in explicit sports skills or physical performance, but may be linked to mental well-being through students’ perceived competence, willingness to participate, and self-acceptance.

Further statistical mediation analysis showed that the sense of belonging in the sports environment partially accounted for the cross-sectional association between physical literacy and mental well-being, with the indirect effect accounting for approximately 46.9% of the total effect. This result suggests that the relationship between physical literacy and mental health is not only directly associated but is also statistically partially manifested as an indirect link formed via emotional experiences and social connections in the sports environment. Viewing this through Self-Determination Theory, students with higher physical literacy are more likely to achieve a sense of competence and participation in PE classes or campus sports activities, and enter peer interactions and group activities with a more open attitude; the satisfaction of autonomy, competence, and relatedness needs forms an important foundation for promoting positive motivation and psychological adaptation (Deci and Ryan, 2000; Standage et al., 2005). Belongingness, as a crucial resource for college adaptation and mental health, has been repeatedly emphasized in studies on college students and student athletes (Storey et al., 2024; Yildirim et al., 2021).

Therefore, the results of this study further imply that the key to promoting mental health through college physical education does not lie solely in increasing students’ exercise volume or physical fitness levels, but more importantly in whether it helps students form positive sports self-cognitions and attain genuine belonging experiences in the sports environment. In other words, physical literacy may provide the cognitive and motivational foundation for students to form positive sports experiences, while the sense of belonging in the sports environment serves as an important psychological channel connecting this foundation with mental health indicators. This also echoes the core philosophy of inclusive physical education: physical education should not merely focus on whether students “complete sports tasks,” but also on whether they feel “I can participate, I am accepted, I belong here” during sports participation. By prioritizing these social and emotional processes in late adolescence, physical educators can foster a baseline of psychological safety that bridges educational environments with adult wellness habits.

### 5.2 The conditional role of exercise groups and sports participation trajectories

This study further examined the moderating role of exercise groups and the interactive effects of sports participation trajectories. The results indicated that exercise groups classified based on the total LSI score did not significantly moderate the relationship between the sense of belonging in the sports environment and mental health, and the differences between the active group and the control group in physical literacy, sense of belonging, and mental health were non-significant. These results suggest that, in the present sample, the “active exercise” status classified solely by the total amount of physical activity may be insufficient to explain differences in students’ mental health levels.

This finding does not negate the positive significance of physical activity for mental health, but rather indicates that the relationship between physical activity and mental health may be jointly affected by factors such as activity intensity, exercise motivation, social support, and participation quality (Han et al., 2025a; Han et al., 2025b; Malagodi et al., 2025). These complexities align with a growing body of evidence, notably the DE-PASS systematic review and meta-analysis, which emphasizes that physical activity behavior and mental health are shaped by a complex interplay of modifiable individual, social, and environmental determinants across school, household, and mixed settings (Ciaccioni et al., 2026). Rather than physical activity volume alone acting as a direct silver bullet, factors such as social connectedness, supportive pedagogy, and structured sports environments operate as secondary modifiable channels. The HCS sensitivity analysis of this study also supported this judgment: when grouped using the total LSI score that includes mild-intensity activities, the proportion of the active exercise group was significantly higher than the HCS grouping result based only on moderate-to-vigorous activity scores. Thus, different scoring methods of the GLTEQ affect the categorization of exercise groups, and when explaining the relationship between physical activity levels and mental health, total activity volume should be distinguished from moderate-to-vigorous physical activity volume (Godin, 2011; Godin and Shephard, 1985).

Furthermore, the interactive association between high school sports participation levels and university sports club participation on physical literacy and sense of belonging in the sports environment did not reach significance. This may be related to the relatively simplified measurement of sports participation experiences in this study. High school sports participation levels and university sports club participation were both expressed as categorical variables, which are difficult to reflect finer details such as years of participation, training frequency, depth of participation, team roles, competition experience, and the quality of club interactions. Therefore, the lack of support for H4 is better understood as: at the current granularity of measurement, sports participation identity alone is insufficient to explain differences in physical literacy and sense of belonging in the sports environment, and it cannot be inferred that early sports experiences or university sports club participation are entirely unimportant.

Overall, the non-significant results of H3 and H4 jointly demonstrate that the sports-related mechanisms of college students’ mental health may not primarily depend on “whether one exercises actively” or “whether one has a sports participation identity,” but more on whether students receive positive self-evaluation, emotional support, and belonging experiences in the sports environment. For college physical education, while increasing sports participation opportunities is important, what is more crucial is to improve the quality of interaction, supportive atmosphere, and psychological safety in sports activities.

### 5.3 Practical implications in the context of national fitness

Based on the era’s opportunity to comprehensively advance inclusive physical education, the empirical findings of this study can provide some references for college physical education reform.

Inclusive physical education research emphasizes that the PE classroom should provide a fair, safe, and dignified participation environment for students with different abilities, physical conditions, and participation experiences (Block and Obrusnikova, 2007; Goodwin and Watkinson, 2000; Pushkarenko et al., 2021). The past evaluation logic that overemphasized sports performance (e.g., heart rate compliance, lap counts) has somewhat ignored those students lingering on the margins of sports venues. If the key to supporting students’ mental health development is not just increasing exercise frequency, then future PE classrooms should lower individuals’ psychological defenses through more group collaboration and peer evaluation rather than simple ranking comparisons, transforming physical literacy into genuine social assets. Furthermore, these findings have important implications for PE teacher education (PETE) programs. Training K-12 and university physical education pre-service teachers to value and construct psychologically safe PE environments—where the focus is shifted from performance pressure to cultivating intrinsic belongingness and physical literacy—is a crucial step in translating policy intentions into inclusive classroom practices.

At the level of management interventions, universities should focus on student groups facing great academic stress (e.g., grades about to face research tasks or employment pressure) by empowering sports clubs to turn sports venues into informal “psychological pressure-relief stations.” This also aligns with the guidance in college physical education and national fitness policies regarding the promotion of healthy lifestyles and lifelong sports awareness among students (State Council of the People’s Republic of China, 2021). To operationalize this, universities are encouraged to integrate sportswear or exercise counseling services into their wellness programs. Specifically, involving sport psychology professionals or mental health counselors directly as collaborators within campus sports and exercise departments can provide students with holistic, non-threatening support. The benign interaction between physical literacy and belongingness suggests that club management should not only focus on how many events are held, but also on how to build a gentle atmosphere that allows students missing early sports trajectories to quickly find a sense of identity. Only when sports become a life experience where students feel safe, respected, and full of meaning can the grand goal of health promotion be truly realized. Moreover, the HCS sensitivity analysis suggests that when evaluating students’ sports participation levels, universities should not judge exercise status merely by total activity frequency, but should further differentiate between mild activities and moderate-to-vigorous activities. Similarly, intervention studies on college students’ physical activity also suggest that exercise frequency, intensity, consistency, and psychological experiences should be focused on simultaneously, rather than judging solely by whether one participates in physical activities (Malagodi et al., 2025; Zhong, 2024). For physical education practice, the key to promoting students’ mental health is not just to increase the number of exercise sessions, but to simultaneously enhance participation quality, classroom interactive experiences, and the level of environmental support.

### 5.4 Limitations of the study

This study still has several limitations. First, this study employed a cross-sectional survey design, meaning all variables were measured at the same time point, and therefore cannot provide evidence of temporal precedence between variables. Although the mediation model was statistically supported, related results should be interpreted as statistical associations rather than causal mechanisms. Future research could utilize longitudinal tracking, cross-lagged models, or physical education intervention designs to further test the dynamic relationships among physical literacy, sense of belonging in the sports environment, and mental health.

Second, the data in this study came primarily from self-report questionnaires, which may be subject to recall bias, social desirability cross-sectional, and common method bias. Although Harman’s single-factor test and CFA results indicated that this study did not have a severe single-method factor issue, this method can only serve as a preliminary diagnosis and still cannot completely rule out the risk of bias brought by homologous measurements (Podsakoff et al., 2003). Crucially, the Average Variance Extracted (AVE) values of the three latent variables (ranging from 0.365 to 0.418) were below the ideal 0.50 threshold, which suggests that the convergent validity of these constructs warrants improvement and caution is advised during data interpretation. In particular, the Sports-Environment Belongingness Scale is a newly developed tool; although its item selection was guided by established theoretical frameworks and expert reviews, its construct validity needs further rigorous validation, expert evaluation, pilot testing on larger independent samples, and measurement equivalence tests across diverse student cohorts.

Third, the measurement of physical activity levels relied heavily on the GLTEQ self-report questionnaire. While this tool has the advantages of being simple, low-burden, and suitable for large-sample surveys, it may still be influenced by recall errors regarding subjects’ weekly exercise frequencies (Godin, 2011; Godin and Shephard, 1985). Importantly, our moderation and sensitivity analyses converted physical activity levels from continuous scoring metrics (LSI/HCS) into categorical groups. While grouping is valuable for defining public health compliance standards, it inherently restricts the preservation of continuous variance, which might have masked more subtle moderating effects. Future research should consider employing exercise behavior as a continuous variable in moderation modeling to cross-verify these patterns. Additionally, this study found that using the LSI and HCS scoring methods yields different exercise grouping results, indicating that the division of exercise groups is heavily influenced by the physical activity algorithm. Therefore, future studies could combine wearable devices, exercise logs, or objective physical activity monitoring data to enhance the accuracy of physical activity measurements.

Furthermore, high school sports participation levels and university sports club participation were both measured using simplified categorical variables, unable to fully reflect quality differences in sports participation experiences. Follow-up studies could further incorporate indicators such as years of participation, training frequency, competition experiences, team roles, club supportive atmospheres, and peer interaction quality to more accurately examine the impact of sports participation trajectories on physical literacy and sense of belonging in the sports environment.

Finally, while the sample size of this study is somewhat large, the sources remained concentrated in specific universities and specific survey contexts. Universities in different regions and at different educational levels may have differences in sports resource allocation, course organization methods, campus sports culture, and student stress levels. Therefore, caution is still needed when generalizing the conclusions of this study to broader college student populations. Future studies could expand sample sources to conduct comparative studies across regions, university types, and major groups to enhance the external validity of the conclusions.

## 6 Conclusion

Based on a sample of 890 college students, this study examined the statistical associations among physical literacy, sense of belonging in the sports environment, exercise groups, and mental health. The results showed that perceived physical literacy was significantly and positively associated with the physical and mental adaptation state reflected by the WHO-5, and the sense of belonging in the sports environment played a significant partial mediating role between the two. This result illustrates that the positive association between physical literacy and mental health is not only reflected at the level of athletic ability or health cognition, but is also statistically partially manifested in association with the students’ sense of acceptance, integration, and psychological safety in PE classes and campus sports activities.

In contrast, exercise groups classified based on total LSI scores did not significantly moderate the relationship between belongingness and mental health, nor did high school sports participation levels and university sports club participation cast a significant interactive association on physical literacy and the sense of belonging in the sports environment. Given the cross-sectional nature of this study, these findings represent statistical correlation pathways rather than causal mechanisms. This suggests that simple exercise volume, early sports participation experiences, or club participation identity are not sufficient to fully explain variations in college students’ mental health; subjective belonging experiences in the sports environment might be a psychological connection mechanism more worthy of attention.

Furthermore, the HCS sensitivity analysis revealed that different physical activity grouping algorithms significantly impact the proportion of active exercise groups. When discussing exercise classes in future studies, overall activity volume should be further distinguished from moderate-to-vigorous physical activity volume. Overall, this study emphasizes that the key to supporting college students’ mental health development through physical education is perhaps not just “getting students to exercise more,” but helping students form a positive psychological experience of “I can participate, I am accepted, I belong here” within the sports environment.

## Data Availability

The original contributions presented in the study are included in the article/Supplementary material, further inquiries can be directed to the corresponding author.
