# Supplementary material for: Physical literacy, sense of belonging in the sports environment, and college students’ mental health: an analysis of relational paths based on differences in exercise groups
Source: Front Psychol. 2026 Aug 4;17:1896855. doi: 10.3389/fpsyg.2026.1896855 (PMC13481310; doi:10.3389/fpsyg.2026.1896855)
Supplement: Supplementary file 1 [file Data_Sheet_1.ZIP › Supplementary Material 2_Variable Codebook.docx]

This codebook provides the variable names, corresponding questionnaire items, variable descriptions, coding schemes, and derived variables used in the dataset. The full questionnaire text is provided in Supplementary Material 1.

## Table S2.1. Original Questionnaire Variables and Coding Scheme

| Variable Name | Questionnaire Item | Variable Description | Coding Scheme |
| --- | --- | --- | --- |
| ID | — | Participant identification number | Numeric identifier assigned to each valid response |
| Q1_Consent | Q1 | Informed consent | A = Agreed to participate; B = Did not agree to participate |
| Q2_Gender | Q2 | Gender | 1 = Male; 2 = Female |
| Q3_Age | Q3 | Age | Continuous variable, measured in years |
| Q4_Grade | Q4 | Year of study | 1 = Freshman; 2 = Sophomore; 3 = Junior; 4 = Senior or above |
| Q5_Major | Q5 | Academic major category | 1 = Humanities / Social Sciences / Management; 2 = Science / Engineering / Agriculture / Medicine; 3 = Arts / Media; 4 = Physical Education-related major |
| Q6_Origin | Q6 | Place of origin | 1 = Urban area, including county towns; 2 = Rural area |
| Q7_Height | Q7 | Height | Continuous variable, measured in centimeters |
| Q8_Weight | Q8 | Weight | Continuous variable, measured in kilograms |
| Q9_Injury | Q9 | Severe injury or medical contraindication for physical activity | A = Yes; B = No |
| Q10_FamilySES | Q10 | Subjective family economic status | 1 = Relatively poor / below average; 2 = Moderate / average; 3 = Relatively good / above average |
| Q11_AcademicStress | Q11 | Academic stress | 1 = Very low; 2 = Low; 3 = Moderate; 4 = High; 5 = Very high |
| Q12_HighSchoolSports | Q12 | High-school sports participation level | 1 = Almost no participation; 2 = Regular PE only with occasional extracurricular exercise; 3 = Frequent extracurricular exercise without systematic specialized training; 4 = School team membership or systematic high-intensity specialized training |
| Q13_CollegeClub | Q13 | University sports club/team participation | 1 = Participated; 2 = Did not participate |
| Q14_PEClass | Q14 | Enrollment in a university PE course during the current semester | 1 = Currently enrolled; 2 = Not currently enrolled |
| Q15_Strenuous | Q15 | Weekly frequency of strenuous physical activity | Continuous variable, times per week |
| Q16_Moderate | Q16 | Weekly frequency of moderate physical activity | Continuous variable, times per week |
| Q17_Mild | Q17 | Weekly frequency of mild physical activity | Continuous variable, times per week |
| Q18_PPLI–Q22_PPLI | Q18–Q22 | Perceived Physical Literacy Instrument items | 1 = Strongly disagree / not at all true; 5 = Strongly agree / completely true |
| Q23_Belong–Q27_Belong | Q23–Q27 | Physical environment belongingness items | 1 = Strongly disagree / not at all true; 5 = Strongly agree / completely true |
| Q28_WHO5–Q32_WHO5 | Q28–Q32 | WHO-5 well-being items | 0 = At no time; 5 = All of the time |

## Table S2.2. Derived Variables

| Derived Variable | Calculation / Definition | Interpretation |
| --- | --- | --- |
| BMI | Q8_Weight / (Q7_Height / 100)^2 | Body mass index calculated from self-reported height and weight |
| PPLI | Mean of Q18_PPLI–Q22_PPLI | Higher scores indicate higher perceived physical literacy |
| Belong | Mean of Q23_Belong–Q27_Belong | Higher scores indicate stronger belongingness in PE classes or campus sports environments |
| WHO5 | Mean of Q28_WHO5–Q32_WHO5 | Higher scores indicate more positive subjective well-being and psychosocial adaptation |
| LSI_total | $\text{9×}\text{Q}\text{1}\text{5}_{\text{S}}\text{trenuous}\text{+5×}\text{Q}\text{1}\text{6}_{\text{M}}\text{oderate}\text{+3×}\text{Q}\text{1}\text{7}_{\text{M}}\text{ild}$ | Godin Leisure-Time Exercise Index including strenuous, moderate, and mild physical activity |
| HCS_mvpa | $\text{9×}\text{Q}\text{1}\text{5}_{\text{S}}\text{trenuous}\text{+5×}\text{Q}\text{1}\text{6}_{\text{M}}\text{oderate}$ | Health Contribution Score based only on strenuous and moderate physical activity |
| LSI_group_total | LSI_total < 14 = insufficiently active; 14 ≤ LSI_total < 24 = moderately active; LSI_total ≥ 24 = active | Physical activity grouping based on total LSI score |
| HCS_group | HCS_mvpa < 14 = insufficiently active; 14 ≤ HCS_mvpa < 24 = moderately active; HCS_mvpa ≥ 24 = active | Sensitivity grouping based on strenuous and moderate physical activity only |
| HS_sport_binary | Q12_HighSchoolSports = 1 or 2 = lower high-school sports participation; Q12_HighSchoolSports = 3 or 4 = higher high-school sports participation | Binary grouping for high-school sports participation level |
| CollegeClub_binary | Q13_CollegeClub = 1 = participated; Q13_CollegeClub = 2 = did not participate | Binary grouping for university sports club/team participation |
