# Supplementary material for: Physical literacy, sense of belonging in the sports environment, and college students’ mental health: an analysis of relational paths based on differences in exercise groups
Source: Front Psychol. 2026 Aug 4;17:1896855. doi: 10.3389/fpsyg.2026.1896855 (PMC13481310; doi:10.3389/fpsyg.2026.1896855)
Supplement: Supplementary file 1 [file Data_Sheet_1.ZIP › Supplementary Material 1_Full Questionnaire.docx]

# Supplementary Material: Questionnaire Text

## Survey on Physical Literacy, Sense of Belonging, and Psychological Adaptation among College Students

### **[Part 1: Research Description and Informed Consent]**

Dear Student,
Thank you for taking the time to participate in this academic survey. This study aims to explore the extracurricular physical activity patterns of contemporary college students and their associations with the sense of belonging in physical education (PE) classes and psychological adaptation.

This survey strictly adheres to academic ethics guidelines. It is completely **anonymous**, and we will not collect any sensitive personal privacy information such as your name, student ID, or phone number. All collected data will be encrypted by the research team and used solely for statistical analysis at the group level for academic publications. We will never disclose any individual information to third parties.

It will take approximately 5 minutes to complete this questionnaire. Your objective and truthful responses are crucial to the scientific validity of this research. Participation in this study is entirely voluntary, and you may unconditionally withdraw at any point during the process. We sincerely appreciate your support and cooperation!

**1. Informed Consent Confirmation: I confirm that I am at least 18 years old, have carefully read and fully understood the research description above, and voluntarily agree to participate in this study. [Single Choice]**

A. Yes, I agree to participate in the survey. *(Skip logic: Proceed to Question 2)*

B. No, I do not agree to participate. *(Skip logic: Terminate survey, do not record data)*

### **[Part 2: Sociodemographic and Baseline Characteristics]**

**Instructions: Please select the option that best fits your actual situation or fill in the specific values.**

**2. Gender: [Single Choice]**
[ ] Male [ ] Female

**3. Actual Age: [Fill-in-the-blank]**
________ years old

**4. Current Grade Level: [Single Choice]**
[ ] Freshman [ ] Sophomore [ ] Junior [ ] Senior or above

**5. Which category does your major belong to? [Single Choice]**
[ ] Humanities / Social Sciences / Management
[ ] Science / Engineering / Agriculture / Medicine
[ ] Arts / Media
[ ] Physical Education (PE) / Sports Major

**6. Origin/Hometown: [Single Choice]**
[ ] Urban (including county towns) [ ] Rural

**7. Height: [Fill-in-the-blank]**
________ cm

**8. Weight: [Fill-in-the-blank]**
________ kg

**9. Do you currently have a severe injury or medical condition for which a doctor has advised “absolute strict prohibition of physical activity”? [Single Choice]**
[ ] A. Yes *(Skip logic: Terminate survey to screen out invalid samples incapable of exercise)*
[ ] B. No

**10. Compared to your peers, your subjective perception of your family’s socioeconomic status is: [Single Choice]**
[ ] Lower / Below average [ ] Moderate / Average [ ] Higher / Above average

**11. Recently, your perceived overall level of academic stress is: [Single Choice]**
[ ] Very low [ ] Low [ ] Moderate [ ] High [ ] Very high

**12. During your high school years (the critical period before entering college), what was your daily extracurricular physical activity involvement? [Single Choice]**
[ ] A. Rarely participated in physical exercise / skipped PE classes due to academics.
[ ] B. Only attended regular PE classes, occasionally exercised in free time.
[ ] C. Frequently exercised in free time but did not receive systematic special training.
[ ] D. Was a school varsity team member or received systematic, high-intensity sports training.

**13. In your current college phase, have you joined any regular sports clubs, organizations, or university/college representative sports teams? [Single Choice]**
[ ] Yes, I have joined

[ ] No, I have not

**14. Are you currently enrolled in a public PE course (compulsory or elective) offered by your university this semester? [Single Choice]**
[ ] Yes, currently taking a PE course

[ ] No, not taking a PE course currently

### **[Part 3: Godin-Shephard Leisure-Time Physical Activity Questionnaire (GLTEQ)]**

**Instructions: Considering a 7-day period (a week), how many times on the average do you do the following kinds of exercise for more than 15 minutes during your free time? Please write the specific number of “times” on the blank line (If zero, please enter “0”).**

**15. STRENUOUS EXERCISE (Heart beats rapidly, sweating heavily):**
*(e.g., running, basketball/football full-court games, High-Intensity Interval Training (HIIT), vigorous swimming, etc.)*
Times Per Week: ________ [Fill-in-the-blank]

**16. MODERATE EXERCISE (Not exhausting, light sweating, slightly faster heartbeat):**
*(e.g., fast walking, jogging, bicycling, recreational volleyball/badminton, easy swimming, etc.)*
Times Per Week: ________ [Fill-in-the-blank]

**17. MILD EXERCISE (Minimal effort, no clear change in heart rate):**
*(e.g., easy walking, morning exercises, yoga stretching, bowling, etc.)*
Times Per Week: ________ [Fill-in-the-blank]

### **[Part 4: Perceived Physical Literacy Instrument (PPLI)]**

**Instructions: Physical literacy encompasses not only motor skills but also cognition and self-management abilities regarding physical activity. Please rate your level of agreement with the following statements. (1 = Strongly Disagree; 5 = Strongly Agree)**

**18.** I am confident in my ability to participate in physical exercise and various sports. (1 2 3 4 5)
**19.** I deeply understand that participating in physical activities is crucial for maintaining and improving my physical and mental health. (1 2 3 4 5)
**20.** I am able to communicate, cooperate, and coordinate well with my peers during physical activities. (1 2 3 4 5)
**21.** I possess the basic knowledge and methods required to independently engage in common physical exercises in my daily life. (1 2 3 4 5)
**22.** I have the intrinsic motivation to consciously integrate physical exercise as a part of my lifestyle. (1 2 3 4 5)

### **[Part 5: Sense of Belonging in Physical Education Settings]**

**Instructions: The following questions aim to understand your inner feelings when participating in college PE courses or campus group sports activities. Please rate based on your actual feelings. (1 = Severely Disagree; 5 = Strongly Agree)**

**23.** During PE classes or campus sports activities, I feel completely integrated into the group. (1 2 3 4 5)
**24.** I feel that the PE teachers and surrounding classmates are willing to accept me and respect my sports performance. (1 2 3 4 5)
**25.** In the PE classroom, I feel I get along very well with surrounding classmates and we generate positive interactions. (1 2 3 4 5)
**26.** When I encounter difficulties in practicing a certain motor skill, I am confident that my classmates or teachers will be happy to help me. (1 2 3 4 5)
**27.** Participating in school sports activities brings me a strong sense of belonging and psychological security. (1 2 3 4 5)

### **[Part 6: WHO-5 Well-Being Index]**

**Instructions: Mental health and positive adaptive capacities are equally our focus. Please reflect carefully on how often you have experienced the following mental states over the last two weeks (14 days). (0 = At no time; 5 = All of the time)**

*Over the last two weeks…*
**28.** I have felt cheerful and in good spirits. (0 1 2 3 4 5)
**29.** I have felt calm and relaxed. (0 1 2 3 4 5)
**30.** I have felt active and vigorous. (0 1 2 3 4 5)
**31.** I woke up feeling fresh and rested. (0 1 2 3 4 5)
**32.** My daily life has been filled with things that interest me. (0 1 2 3 4 5)

**[Conclusion]**
This is the end of the questionnaire. Thank you sincerely for your objective and patient responses. Your data are of immense value to our research! Wishing you success in your studies and excellent physical and mental health!
